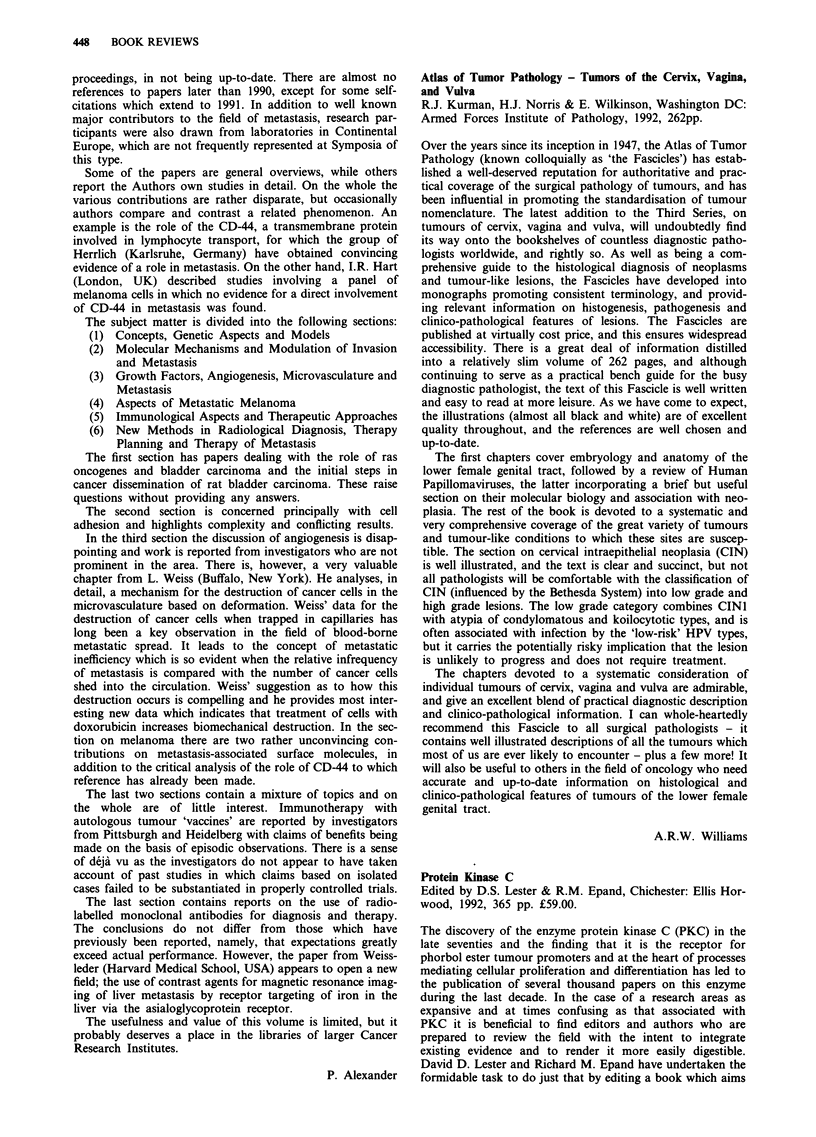# Atlas of Tumor Pathology - Tumors of the Cervix, Vagina, and Vulva

**Published:** 1993-08

**Authors:** A.R.W. Williams


### Atlas of Tumor Pathology – Tumors of the Cervix, Vagina, and Vulva

R.J. Kurman, H.J. Norris & E. Wilkinson, Washington DC: Armed Forces Institute of Pathology, 1992, 262pp.

Over the years since its inception in 1947, the Atlas of Tumor Pathology (known colloquially as 'the Fascicles') has established a well-deserved reputation for authoritative and practical coverage of the surgical pathology of tumours, and has been influential in promoting the standardisation of tumour nomenclature. The latest addition to the Third Series, on tumours of cervix, vagina and vulva, will undoubtedly find its way onto the bookshelves of countless diagnostic pathologists worldwide, and rightly so. As well as being a comprehensive guide to the histological diagnosis of neoplasms and tumour-like lesions, the Fascicles have developed into monographs promoting consistent terminology, and providing relevant information on histogenesis, pathogenesis and clinico-pathological features of lesions. The Fascicles are published at virtually cost price, and this ensures widespread accessibility. There is a great deal of information distilled into a relatively slim volume of 262 pages, and although continuing to serve as a practical bench guide for the busy diagnostic pathologist, the text of this Fascicle is well written and easy to read at more leisure. As we have come to expect, the illustrations (almost all black and white) are of excellent quality throughout, and the references are well chosen and up-to-date.

The first chapters cover embryology and anatomy of the lower female genital tract, followed by a review of Human Papillomaviruses, the latter incorporating a brief but useful section on their molecular biology and association with neoplasia. The rest of the book is devoted to a systematic and very comprehensive coverage of the great variety of tumours and tumour-like conditions to which these sites are susceptible. The section on cervical intraepithelial neoplasia (CIN) is well illustrated, and the text is clear and succinct, but not all pathologists will be comfortable with the classification of CIN (influenced by the Bethesda System) into low grade and high grade lesions. The low grade category combines CIN1 with atypia of condylomatous and koilocytotic types, and is often associated with infection by the 'low-risk' HPV types, but it carries the potentially risky implication that the lesion is unlikely to progress and does not require treatment.

The chapters devoted to a systematic consideration of individual tumours of cervix, vagina and vulva are admirable, and give an excellent blend of practical diagnostic description and clinico-pathological information. I can whole-heartedly recommend this Fascicle to all surgical pathologists – it contains well illustrated descriptions of all the tumours which most of us are ever likely to encounter – plus a few more! It will also be useful to others in the field of oncology who need accurate and up-to-date information on histological and clinico-pathological features of tumours of the lower female genital tract.

A.R.W. Williams